# Excess pancreatic cancer risk due to smoking and modifying effect of quitting smoking: The Multiethnic Cohort Study

**DOI:** 10.1007/s10552-023-01811-x

**Published:** 2023-11-04

**Authors:** David Bogumil, Daniel Stram, Dale L. Preston, Stephen J. Pandol, Anna H. Wu, Roberta McKean-Cowdin, David V. Conti, Veronica Wendy Setiawan

**Affiliations:** 1 Department of Preventive Medicine, Keck School of Medicine, University of Southern California, 1450 Biggy Street, Room 1517C, Los Angeles, CA 90033 USA; 2 Epidemiology Program, University of Hawaii Cancer Center, Honolulu, HI USA; 3 Hirosoft International, Eureka, CA USA; 4 Division of Gastroenterology, Department of Medicine, Cedars-Sinai Medical Center, Los Angeles, CA USA; 5 Department of Veterans Affairs, Los Angeles, CA USA; 6 Center for Genetic Epidemiology, Keck School of Medicine, University of Southern California, Los Angeles, CA USA; 7 Norris Comprehensive Cancer Center, Keck School of Medicine, University of Southern California, Los Angeles, CA USA

**Keywords:** Pancreas, Cancer, Smoking, Multiethnic

## Abstract

**Purpose:**

Risk factors for pancreatic cancer include racial/ethnic disparities and smoking. However, risk trajectories by smoking history and race/ethnicity are unknown. We examined the association of smoking with pancreatic cancer by race/ethnicity to generate age-specific incidence estimates by smoking history.

**Methods:**

We modeled pancreatic cancer incidence by race/ethnicity, age, pack-years, and years-quit using an excess relative risk model for 182,011 Multiethnic Cohort participants. We tested heterogeneity of smoking variables and pancreatic cancer by race/ethnicity and predicted incidence by smoking history.

**Results:**

We identified 1,831 incident pancreatic cancer cases over an average 19.3 years of follow-up. Associations of pack-years (*p* interaction by race/ethnicity = 0.41) and years-quit (*p* interaction = 0.83) with pancreatic cancer did not differ by race/ethnicity. Fifty pack-years smoked was associated with 91% increased risk (95% CI 54%, 127%) relative to never smokers in the combined sample. Every year quit corresponded to 9% decreased excess risk (95% CI 2%, 15%) from pack-years smoked. Differences in baseline pancreatic cancer risk across racial/ethnic groups (*p* < 0.001) translated to large differences in risk for smokers at older ages across racial/ethnic groups (65–122 cases per 100,000 at age 70).

**Conclusion:**

Smoking pack-years were positively associated with elevated pancreatic cancer risk. Predicted risk trajectories showed a high impact of smoking cessation at < 65 years. Although we did not identify significant heterogeneity in the association of pack-years or years quit with pancreatic cancer risk, current smoker risk varied greatly by race/ethnicity in later life due to large differences in baseline risk.

**Supplementary Information:**

The online version contains supplementary material available at 10.1007/s10552-023-01811-x.

## Introduction

In 2023, pancreatic cancer is projected to account for 64,000 cancer cases and 50,000 deaths in the United States [1]. Pancreatic cancer is the third leading cause of cancer death and is projected to be the second leading cause by 2040 [1, 2]. Because there are no regular forms of screening, pancreatic cancer is commonly diagnosed at a late stage, making successful treatment difficult. Accordingly, five-year survival is low at 12% [1, 3].

The burden of pancreatic cancer differs across ethnic/racial groups, with the highest incidence among non-Hispanic Blacks, Japanese Americans, non-Hispanic whites, and Hispanics [1, 4]. Risk factors for pancreatic cancer include obesity [5, 6], type 2 diabetes [5, 7], family history of pancreatic cancer [5], pancreatitis [8], and common genetic variants [9]. Smoking is one of the strongest and most prevalent risk factors, conferring 70–80% increased risk relative to never smokers [5, 10–12]. Smoking is a particularly important risk factor because it is modifiable—risk can be largely attenuated by quitting smoking [5, 11]. Variation in associations of smoking by race/ethnicity are expected given known differences in metabolism, types of cigarettes smoked (menthol/nonmenthol), and smoking duration prior to cessation among racial/ethnic groups [13–15].

Most prior studies evaluating the impact of smoking on risk of pancreatic cancer only report relative measures of risk, although incidence by smoking history is critical to understand and communicate this exposure’s effect. Exposure-specific incidence rates are valuable to communicate disease risk to patients in the clinical setting and are more easily comparable to age-specific incidence rates commonly reported in public health resources such as the National Cancer Institute’s Surveillance, Epidemiology, and End Results (SEER) program.

In this study, we used Poisson regression in the form of an excess relative risk (ERR) model to estimate the incidence of pancreatic cancer by smoking patterns and race/ethnicity in the Multiethnic Cohort (MEC) study. The ERR model separates baseline risk and excess risk from an exposure of interest, allowing us to test sets of modifying variables on an exposure and estimate incidence. Using repeated measures of smoking in this prospective cohort, we examined modification of the smoking–pancreatic cancer association by smoking cessation, smoking intensity, and race/ethnicity to present detailed incidence estimates across these variables by age.

## Materials and methods

### Study population

We used MEC data to examine the associations of pack-years smoked and years-quit with pancreatic cancer risk. MEC is a population-based prospective study composed of > 215,000 men and women living in California (mainly Los Angeles County) or Hawaii at enrollment. Participants were identified using records from the Department of Motor Vehicles, Health Care Financing Administration files, and voter registration lists. Detailed information on enrollment, data collection, and follow-up have been previously described [16]. Briefly, participants were enrolled in 1993–1996, when they were 45–75 years of age, and self-identified as one of five major racial/ethnic groups (African American, European American, Japanese American, Latin American, and Native Hawaiian). Information on baseline characteristics, such as demographics, diet, anthropometric measures, and lifestyle factors, were collected via self-administered questionnaires through mail.

Our analysis was restricted to 182,011 participants who self-reported at baseline into one of the five major ethnic/racial groups; had valid/non-missing baseline responses for type 2 diabetes (T2D) and body mass index (BMI); had no history of pancreatic cancer; had complete responses for smoking status (never, past, current); and, for current or past smokers, had complete responses for number of years smoked, average number of cigarettes smoked per day, and years since quit. Self-reported baseline T2D status and BMI were used for adjustment in all models.

#### Smoking assessment

Smoking was assessed by self-report on both baseline and follow-up questionnaires. Smoking status (ever/never) was assessed with the question, “Have you ever smoked a total of 20 or more packs of cigarettes in your lifetime?” Pack-years were calculated using self-reported cigarettes smoked per day and years smoked, assessed with the questions: “What is the total number of years you smoked?” and “What is the average number of cigarettes that you smoked per day?” Years-quit was assessed with the question: “If you quit smoking, how long ago did you quit?” A follow-up survey distributed 10 years after baseline asked the same questions and provided updated information on quitting and pack-years. Follow-up survey responses were used to predict pack-years among current smokers at last contact.

#### Pancreatic cancer case ascertainment

Incident cases of pancreatic cancer were identified through annual linkage to the California and Hawaii State Cancer Registry system, which is part of the SEER program. National Death Index and state death certificate files were used to obtain additional information on vital status and cause of death. Cases were defined using ICD-O-3 site codes C25.0–C25.9. There were 1,831 pancreatic cancer cases identified between cohort entry and time of censorship on 31 December 2017. This study was approved by the Institutional Review Boards of the University of Hawaii and University of Southern California.

### Statistical analysis

#### Excess relative risk model

Associations of smoking and smoking cessation with risk of pancreatic cancer were estimated using Poisson regression with an ERR model. This model has previously been implemented in the MEC to measure excess risk of lifetime smoking exposure in association with lung cancer incidence [17]. In our analysis, the ERR model estimated incidence of pancreatic cancer as a function of age, race/ethnicity, sex, T2D, BMI, pack-years smoked, cigarettes smoked per day, and years-quit smoking. We modeled the hazard of pancreatic cancer at age *t* as:

$$h\left(t\right)=\text{baseline}\left(t;{\frac{{\text{race}}}{{\text{ethnicity}}}}; {\text{sex; T}}2 {\text{D; BMI}}\right)(1+{\text{ERR}}({\text{pkyrs}}(t),{\text{years quit}}(t),{\text{CPD}}))$$

In this model, the baseline term was the model component for the hazard (i.e., age-specific incidence rate) among never smokers from racial/ethnic group *j*. This baseline component was assumed to take the loglinear form $\text{baseline}\left(t\right)=\text{exp}({a}_{j}+b\text{log}\left(t\right))$, as a function of age *t* and race/ethnicity *j*. This model was a simple polynomial $\text{exp}\left({a}_{j}\right){t}^{b}$, as in the classic Armitage Doll model. We expressed age in units of 70 years so that $\text{exp}({a}_{j})$ was the estimate of risk for a 70-year-old non-smoker. T2D and BMI were based on self-report at baseline. The ERR term in the model was in the form:

$$\text{ERR}\left(t\right)=c \text{pkyrs}\left(t\right)\text{exp}(d \text{years}\_\text{quit}\left(t\right) \text{e} \text{log}(\text{CPD}/20))$$

Years-quit and log-transformed cigarettes smoked per day were allowed to modify the linear association of pack-years on the ERR term. The loglinear part of the ERR term was a dose modifier in that it had no meaning if pack-years was zero.

To estimate parameters *a–e* in the model, we developed a finely stratified person-years table, with case counts, stratified on race/ethnicity and 5-year intervals of age, and age-dependent variables of pack-years and years-quit. Exploiting the link (Holford, Laird Oliver) between the likelihood for a piecewise exponential model and Poisson regression for survival analysis [18], we created tables and fit the models above by maximum likelihood to table counts using Epicure software [19].

#### Pack-years estimation

For ex-smokers at baseline, pack-years was treated as a fixed (not time-dependent) variable. For current smokers, however, pack-years continued to accumulate, based on the assumption that participants who smoked at baseline would continue to smoke until censorship. To estimate pack-years and years-quit among current smokers following last contact, we used data from 11,630 baseline smokers who returned follow-up questionnaires to model quitting behavior. This model was used to estimate smoking exposure beyond last completed follow-up questionnaire. Details of this model are in the Supplementary Material of the MEC publication on excess lung cancer risk among smokers [17]. Briefly, pack-years among current smokers beyond the last questionnaire were estimated by fitting a survival model of quitting behavior, with the “hazard” of quitting being a linear function of race/ethnicity, sex, age at entry, time on study, and cigarettes smoked per day among ex-smokers. Using the hazard function from this model, in conjunction with participant characteristics used to predict quitting behavior, we estimated pack-years and years-quit for participants who self-reported as smokers at the last questionnaire. Among those who returned the follow-up questionnaire, the most recent information on smoking behavior was used to estimate pack-years (e.g., questionnaire 2), allowing direct calculation of pack-years between questionnaires in cases where the follow-up survey was returned.

#### ERR model fit

When fitting the ERR model to predict pancreatic cancer risk, parameters *a–e* in the baseline term and ERR terms were estimated simultaneously using all participants. Parameter estimates of the fit model are in Supplementary Tables 1 and 2. Wald tests and two-sided p-values were calculated for model estimates, and likelihood ratio tests (LRT) were used to test heterogeneity of estimates by race/ethnicity. In addition to our main results, we conducted a stratified analysis by sex to estimate sex-specific rates by smoking history. We also conducted a sensitivity analysis adjusting for alcohol consumption, although we report models without this variable since it did not change the associations for smoking-related variables.

## Results

### Sample characteristics

The final analysis included 182,011 participants, with an average follow-up time of 19.3 years, representing 3,519,587 person-years. Over the study duration there were 1,831 incident cases of pancreatic cancer, with most cases among Japanese Americans (*n* = 648), followed by European Americans (*n* = 357), Latin Americans (*n* = 342), African Americans (*n* = 322), and Native Hawaiians (*n* = 162) (Table 1). The highest proportion of never-smokers was among Japanese Americans (50.8%) and Latin Americans (50.6%). Among current and former smokers at baseline, European Americans (36.4%) and Native Hawaiians (27.7%) had the greatest number of individuals smoking > 20 cigarettes per day. European Americans had the greatest number of former smokers (43.8%), with all other racial/ethnic groups having a similar proportion of former smokers (35.1%–38.2%). Among former smokers, Japanese Americans (52.0%) and European Americans (51.2%) had the largest proportion with ≥ 15 years-quit, followed by Latin Americans (45.3%), Native Hawaiians (43.4%), and African Americans (39.2%). Pancreatic cancer incidence rates age-adjusted to the 2000 US Standard Population showed the highest incidence among Native Hawaiians (50.4 per 100,000), followed by African Americans (38.4 per 100,000), Japanese Americans (34.9 per 100,000), European Americans (25.8 per 100,000), and Latin Americans (25.5 per 100,000) (Table 2).

**Table 1:** Baseline characteristics of study population by race/ethnicity

	Overall (*n* = 182,011)		African American (*n* = 30,169)		European American (*n* = 45,594)		Japanese American (*n* = 52,866)		Latin American (*n* = 40,138)		Native Hawaiian (*n* = 13,244)	
	*n*	%	*n*	%	*n*	%	*n*	%	*n*	%	*n*	%
Pancreatic cancer cases	1,831	1.0	322	1.0	357	0.8	648	1.2	342	0.8	162	1.2
Age at cohort entry (years)												
< 50	30,942	17.0	4,423	14.7	9,398	20.6	8,412	15.9	4,936	12.3	3,773	28.5
50–54	27,466	15.1	4,238	14.0	7,881	17.3	7,001	13.2	5,682	14.2	2,664	20.1
55–59	29,052	16.0	4,487	14.9	6,891	15.1	6,738	12.7	8,856	22.1	2,080	15.7
60–64	31,298	17.2	4,200	13.9	7,036	15.4	8,792	16.6	9,339	23.3	1,931	14.6
65–69	31,752	17.4	6,224	20.6	6,985	15.3	10,457	19.8	6,517	16.2	1,569	11.8
≥ 70	31,501	17.3	6,597	21.9	7,403	16.2	11,466	21.7	4,808	12.0	1,227	9.3
Sex												
Female	100,006	54.9	19,240	63.8	24,627	54.0	27,918	52.8	20,758	51.7	7,463	56.4
Male	82,005	44.9	10,929	44.9	20,967	44.9	24,948	44.9	19,380	44.9	5,781	44.9
Diabetes												
No	160,837	88.4	25,442	84.3	42,936	94.2	47,313	89.5	33,862	84.4	11,284	85.2
Yes	21,174	11.6	4,727	15.7	2,658	5.8	5,553	10.5	6,276	15.6	1,960	14.8
BMI (kg/m^2^)												
< 25	75,888	41.7	8,095	26.8	21,018	46.1	31,884	60.3	11,374	28.3	3,517	26.6
25–29	69,853	38.4	12,357	41.0	16,554	36.3	17,257	32.6	18,707	46.6	4,978	37.6
≥ 30	36,270	19.9	9,717	32.2	8,022	17.6	3,725	7.0	10,057	25.1	4,749	35.9
Smoking history												
Never	82,403	45.3	11,844	39.3	18,121	39.7	26,859	50.8	20,309	50.6	5,270	39.8
Quit < 20 pack-years	52,453	28.8	9,191	30.5	13,511	29.6	14,079	26.6	12,074	30.1	3,598	27.2
Quit ≥ 20 pack-years	17,904	9.8	2,319	7.7	6,481	14.2	5,701	10.8	2,028	5.1	1,375	10.4
Current < 20 pack-years	15,808	8.7	4,474	14.8	2,735	6.0	2,982	5.6	4,067	10.1	1,550	11.7
Current ≥ 20 pack-years	13,443	7.4	2,341	7.8	4,746	10.4	3,245	6.1	1,660	4.1	1,451	11.0
Cigarettes per day^a^												
≤ 10	40,976	41.1	9,556	52.1	7,621	27.7	8,341	32.1	12,733	64.2	2,725	34.2
11–20	34,829	35.0	6,384	34.8	9,847	35.8	10,657	41.0	4,902	24.7	3,039	38.1
> 20	23,803	23.9	2,385	13.0	10,005	36.4	7,009	27.0	2,194	11.1	2,210	27.7
Time since quit^b^												
≤ 5 years	13,741	19.5	2,886	25.1	3,646	18.2	3,148	15.9	2,971	21.1	1,090	21.9
6–15 years	23,028	32.7	4,107	35.7	6,102	30.5	6,351	32.1	4,743	33.6	1,725	34.7
> 15 years	33,588	47.7	4,517	39.2	10,244	51.2	10,281	52.0	6,388	45.3	2,158	43.4

Diabetes, BMI, and smoking history were assessed by self-report at baseline

^a^Among current and former smokers at baseline

^b^Among former smokers at baseline

**Table 2:** Age-adjusted incidence rates of pancreatic cancer

Race/ethnicity	*n*	Cases	Person-years	Incidence rate per 100,000^a^	Rate ratio
African American	30,169	322	538,941	38.36 (44.97, 32.72)	1 (ref)
European American	45,594	357	885,217	25.84 (29.01, 23.01)	0.67 (0.65, 0.69)
Japanese American	52,866	648	1,051,121	34.92 (38.17, 31.94)	0.91 (0.89, 0.93)
Latin American	40,138	342	792,505	25.51 (28.94, 22.49)	0.66 (0.64, 0.69)
Native Hawaiian	13,244	162	251,803	50.39 (59.95, 42.35)	1.31 (1.28, 1.34)

^a^Estimated using observed incidence rates, standardized to the 2000 US Standard Population, truncated at 45 years old

### Racial/ethnic heterogeneity of smoking on risk

In our primary analysis of differing associations of smoking variables with pancreatic cancer risk by race/ethnicity, there was no significant difference in the association between pack-years smoked and pancreatic cancer risk across racial/ethnic groups [*p* LRT = 0.41 on 4 degrees of freedom (df)] (Supplementary Table 1). Additionally, we found no significant difference across racial/ethnic groups in the modifying effect of quitting on the primary association of pack-years (*p* LRT = 0.83, 4 df) or cigarettes smoked per day (*p* LRT = 0.98, 4 df) with pancreatic cancer risk.

Although there was no significant difference in estimates of pack-years by race/ethnicity, the strongest association between smoking and pancreatic cancer risk was observed among Japanese Americans (ERR = 1.31; 95% CI 0.62, 2.01, for current smokers with 50 pack-years smoked relative to never smokers), followed by African Americans (ERR = 1.29; 95% CI 0.32, 2.25), Native Hawaiians (ERR = 0.79; 95% CI − 0.29, 1.86), European Americans (ERR = 0.68; 95% CI 0.01, 1.35), and Latin Americans (ERR = 0.17; 95% CI − 0.59, 0.94). Since these terms represent excess risk due to the exposure beyond baseline risk, relative risk can be considered as 1 + ERR. Thus, 50 pack-years smoked among Japanese Americans was associated with 2.31 (1 + ERR = 1 + 1.31) times the pancreatic cancer risk relative to a never smoker.

The modifying variable of pack-years smoked (years-quit) must be transformed through exponentiation for a more intuitive interpretation of results since it is modeled as a loglinear term. For example, we found the ethnic-specific effect modifier of quitting smoking was only significant among Japanese Americans, with a modifying effect of e^(−0.0669)^ = 0.94 (95% CI 0.88, 0.99)—meaning every year quit smoking corresponded to 0.94-times (or 6.5% decreased) excess risk. While statistically significant, Japanese Americans had the smallest effect modification per year quit, followed by Latin Americans (1 − e^(−0.0858)^ = 8.2% decreased effect of past smoking per year quit), European Americans (1 − e^(−0.1163)^ = 11.0%), African-Americans (1 − e^(−0.1230)^ = 11.6%), and Native Hawaiians (1 − e^(−0.1325)^ = 12.4%).

### Pack-years, years quit, and risk of pancreatic cancer

Because there was no significant difference in associations of pack-years, years-quit, and cigarettes smoked per day with pancreatic cancer risk across racial/ethnic groups, we simplified the excess risk model by fitting a single smoking ERR term for these variables (Supplemental Table 2). In this model, 50 pack-years smoked was associated with 91% increased (95% CI 0.54, 1.27; *p* < 0.001) pancreatic cancer risk relative to a never smoker (as described above, this is the same as HR = 1.91). Smoking cessation significantly modified pack-years, where every year quit corresponded to a 1 − e^(−0.0933)^ = 8.9% (95% CI 2.4%, 15.0%; *p* = 0.008) decreased excess risk of prior smoking.

### Cancer incidence by smoking history

**Fig. 1:**
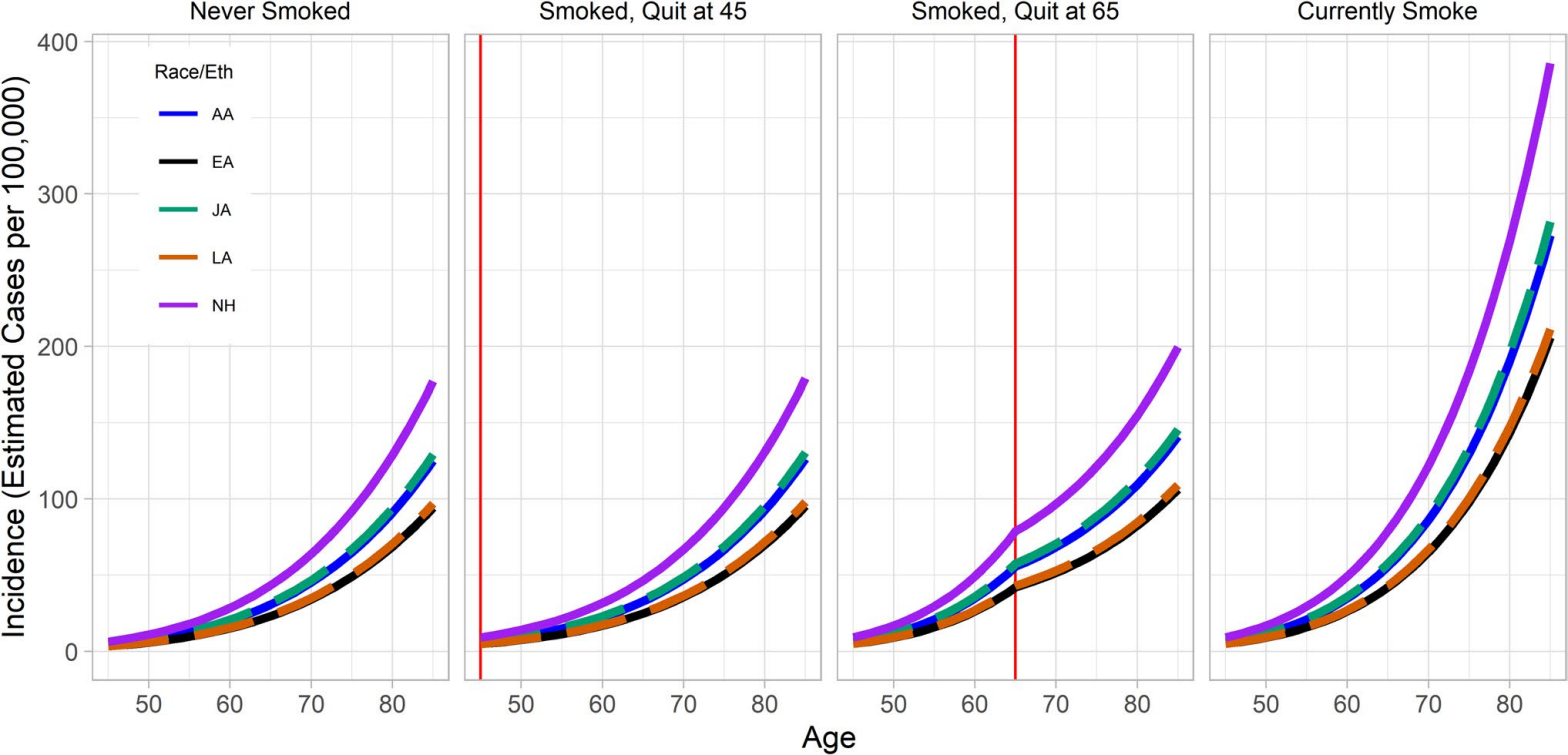
Predicted risk trajectories given differing smoking histories. Risk, in cases per 100,000 (*y*-axis), is plotted as a function of age (*x*-axis), pack-years, and years-quit. The vertical red line indicates modeled age at smoking cessation. To estimate risk in the combined sample, we used a simplified model (Supplementary Table 2)

Although we did not observe significant ethnic heterogeneity of disease risk for pack-years smoked, years-quit, or cigarettes smoked per day, significant differences in baseline risk across groups translated to differing risk based on smoking patterns over a lifetime. To illustrate these differences in pancreatic cancer risk by race/ethnicity, we modeled results in Supplementary Table 2 to estimate incidence of pancreatic cancer across various smoking patterns, under the assumption that participants who smoked began at 20 years old (Fig. 1). Among never smokers, Native Hawaiians had the highest risk of pancreatic cancer at age 70 years (64 cases per 100,000 person-years), compared to other groups that had rates ranging from 34 per 100,000 among Latinos to 47 per 100,000 among Japanese Americans.

This pattern changed when we focused on participants with a smoking history. Among current smokers, risk was consistently highest among Native Hawaiians (122 cases per 100,000 at age 70 years), followed by Japanese Americans (89 per 100,000), African Americans (86 per 100,000), Latin Americans (67 per 100,000), and European Americans (65 per 100,000). Smoking cessation at earlier ages lessened this discrepancy in incidence rates, with early cessation greatly reducing differences. There was no significant difference in the pack-years association with pancreatic cancer risk by sex (*p* = 0.43, Supplemental Table 3). Sex-specific rate curves are in Supplemental Figs. 1 and 2.

## Discussion

Using an ERR model, we found a significant excess risk of pancreatic cancer from smoking and a significant reduction in this excess risk with years-quit smoking. The ERR model allowed us to separate baseline risk and excess risk of smoking into two terms, where baseline risk was only dependent on race/ethnicity, T2D, BMI, sex, and age [17, 18]. This model fit ensured that risk among current and past smokers did not decrease below that of never smokers, a characteristic of this exposure we can safely consider to be true. Additionally, in contrast to Cox regression, which is semi-parametric and does not allow estimates of incidence, our ERR model allowed us to present risk of pancreatic cancer over adult life, given various hypothetical smoking exposure configurations, to better understand the benefit of smoking cessation at different ages. We found that increased pancreatic cancer risk among those who accumulated 30 pack-years but quit early in life (e.g., at < 50 years old) was not dramatically higher than that of never smokers. This is likely because pancreatic cancer risk drops considerably after 10 years of smoking cessation, as we observed in our results as well as in prior studies [12]. This reduction in pancreatic cancer risk among those who quit smoking early in life, prior to the steep increased pancreatic cancer risk due to age, was more drastic relative to those who quit smoking at older ages.

Prior studies show an association between pack-years of smoking and pancreatic cancer risk similar to our findings in a multiethnic population. Pancreatic Cancer Case–Control Consortium data show that > 40 years of smoking is associated with more than two-fold increased risk of pancreatic cancer [OR = 2.10 95% CI 1.58, 2.78], while those who quit smoking 15–20 years ago have a odds ratio of 1.12 (95% CI 0.86, 1.44) and those who quit smoking 20–30 years ago have a odds ratio of 0.98 (95% CI 0.77, 1.23) [20]. Similar results were observed in the EPIC cohort and Pancreatic Cancer Cohort Consortium for years smoked [11, 21], although these studies showed a faster reduction in pancreatic cancer risk among past smokers, with risk returning to that of never smokers after 5–15 years-quit.

Only two studies have attempted to measure the association of pack-years and modifying effects of other smoking parameters on pancreatic cancer risk in a joint model [11, 22]. Schulte et al. used logistic regression and mean-centered smoking variables with an interaction variable for ever/never smoking status [22]. This process estimates smoking risk relative to average smokers [23]. Each year-quit was associated with 4% reduced odds of pancreatic cancer relative to an average smoker [22]. This interpretation differs from ours in that it is relative to an average smoker, whereas our estimate of years quit represents a modifying effect (coefficient) on the pack-years association. More similar to our analysis, Lynch et al. implemented an excess odds ratio model in Epicure using the Pancreatic Cancer Cohort Consortium [11]. In this model, the modifying effect of pack-years only included cigarettes per day, while the modifying effect of years-quit was not estimated.

Our results of homogeneous associations of smoking with pancreatic cancer risk across multiple racial/ethnic groups are supported by a prior MEC analysis using Cox regression [5]. In addition, prior analyses have found homogeneous smoking associations by race/ethnicity for breast cancer but not lung cancer [17, 24]. Additional research on the tobacco–pancreatic cancer mechanism is needed to determine if there are heterogeneous associations by race/ethnicity. Possible mechanisms may be indirect exposure of carcinogenic compounds through the blood stream or bile duct [12, 25] or reduced gastric volume and bile salt reflux, which may damage the main pancreatic duct and lead to cancer [26]. However, these mechanisms are still likely affected by race/ethnicity-specific differences in types of cigarettes smoked, metabolism, and inhalation behavior [13, 15, 17, 27].

The alternative etiologic hypothesis, pancreas metal accumulation from smoking [28, 29] may be less affected by metabolic differences between ethnic/racial groups. However, if a true difference in smoking associations by race/ethnicity exists, limited power in our analysis may have resulted in low power (type 2 error) to detect ethnic/racial differences. This explanation may be supported by our observation of large differences in estimates of increased risk from pack-years smoked among racial/ethnic groups.

Our study has several strengths. This large prospective study was composed of a racially and ethnically diverse population that includes understudied groups in pancreatic cancer research. Additionally, we used an alternative modeling technique to estimate pancreatic cancer incidence by smoking history, which has not yet been done in any population. The main limitation of this study is the reliance on self-reported smoking and quitting behavior among participants. Self-reported smoking measures, which were used to calculate pack-years, may not necessarily correspond to actual cigarette carcinogen exposure levels, as self-reported cigarettes smoked is associated with differing nicotine metabolite levels [30]. Additionally, since we estimated pack-years among last reported current smokers using a survival quitting model, there is uncertainty with this estimation that cannot be accounted for in the main ERR model, although prior use of this quitting model in the MEC shows a strong ability to predict lung cancer risk [17]. Self-report of smoking also contains some degree of information bias. However, the prior MEC analysis of smoking and lung cancer found significant associations between urinary total nicotine equivalents and self-reported cigarettes smoked per day [17], supporting validity of the self-report measure. In the case of underreporting of smoking duration or intensity, our ERR estimates will be biased toward the null, suggesting a larger true association.

In conclusion, we characterized the association between smoking parameters and risk of pancreatic cancer. We determined that the modifying effect of quitting and pack-years accumulated did not differ by race/ethnicity. In our multiethnic population, the modifying effect of quitting smoking appeared greatest prior to entering age groups with high incidence of pancreatic cancer, even after accumulation of > 20 pack-years of smoking. This information may be used in risk communication to middle-age current smokers to encourage smoking cessation. Given the strength of smoking as a risk factor and ubiquity of smoking cessation, more studies using repeated measures of smoking behaviors in joint models are needed to estimate the modifying effect of smoking cessation on pancreatic cancer risk.

### Supplementary Information

Below is the link to the electronic supplementary material.

Supplementary file1 (DOCX 403 KB)

## Data Availability

The individual-level datasets are not available due to privacy restrictions but may be obtained through a MEC data request application (https://www.uhcancercenter.org/for-researchers/mec-data-sharing). The tabulated data used in model fitting for the current study are available from the corresponding author on reasonable request.
