# Supplementary material for: Excess pancreatic cancer risk due to smoking and modifying effect of quitting smoking: The Multiethnic Cohort Study
Source: Cancer Causes Control. 2023 Nov 4;35(3):541–8. doi: 10.1007/s10552-023-01811-x (PMC10838846; doi:10.1007/s10552-023-01811-x)
Supplement: Supplementary file 1 — Supplementary file1 (DOCX 403 KB) [file 10552_2023_1811_MOESM1_ESM.docx]

**Supplementary Tables**

**Supplementary Table 1 Model Statistics for Ethnic-Specific Smoking Associations**

| **Parameter** | **Beta Coefficient** | **Standard Error** | **Test Statistic** | **p-value^a^** | **p LRT^b^** |
| --- | --- | --- | --- | --- | --- |
| **Log-linear Term 1** |  |  |  |  |  |
| Constant | -8.2710 | 0.1815 | -45.5800 | <0.001 |  |
| Sex |  |  |  |  |  |
| Male | ref |  |  |  |  |
| Female | -0.0897 | 0.0493 | -1.8180 | 0.0404 |  |
| Diabetes |  |  |  |  |  |
| No | ref |  |  |  |  |
| Yes | 0.2497 | 0.0704 | 3.5460 | <0.001 |  |
| Race/Ethnicity |  |  |  |  | <0.001 |
| African American | ref |  |  |  |  |
| Native Hawaiian | 0.3846 | 0.1231 | 3.1240 | 0.0018 |  |
| Latin American | -0.1807 | 0.1010 | -1.7880 | 0.0738 |  |
| Japanese American | 0.0754 | 0.0955 | 0.7893 | 0.4300 |  |
| European American | -0.1648 | 0.1010 | -1.6320 | 0.1030 |  |
| Log(age/70) | 5.2460 | 0.2236 | 23.4700 | <0.001 |  |
| BMI | 0.0198 | 0.0055 | 3.5870 | <0.001 |  |
| **Linear Term 1** |  |  |  |  |  |
| Pack-Years Smoked |  |  |  |  |  |
| African American*(pack-years/50) | 1.2860 | 0.4911 | 2.6200 | 0.0088 | 0.4100 |
| Native Hawaiian*(pack-years/50) | 0.7857 | 0.5486 | 1.4320 | 0.1520 |  |
| Latin American*(pack-years/50) | 0.1737 | 0.3898 | 0.4455 | 0.6556 |  |
| Japanese American*(pack-years/50) | 1.3140 | 0.3550 | 3.7020 | <0.001 |  |
| European American*(pack-years/50) | 0.6816 | 0.3428 | 1.9880 | 0.0468 |  |
| **Log-linear Term 2** |  |  |  |  |  |
| Years Quit Smoking |  |  |  |  |  |
| African American*(year-quit) | -0.1230 | 0.0815 | -1.5100 | 0.1310 | 0.8281 |
| Native Hawaiian*(year-quit) | -0.1325 | 0.1837 | -0.7209 | 0.4710 |  |
| Latin American*(year-quit) | -0.0858 | 0.2762 | -0.3106 | 0.7558 |  |
| Japanese American*(year-quit) | -0.0669 | 0.0297 | -2.2510 | 0.0244 |  |
| European American*(year-quit) | -0.1163 | 0.1195 | -0.9727 | 0.3310 |  |
| Cigarettes Smoked per Day |  |  |  |  |  |
| African American*log(CPD/20) | -0.5517 | 0.4929 | -1.1190 | 0.2630 | 0.9832 |
| Native Hawaiian*log(CPD/20) | -0.2756 | 1.3040 | -0.2114 | 0.8321 |  |
| Latin American*log(CPD/20) | -1.1080 | 2.1410 | -0.5175 | 0.6045 |  |
| Japanese American*log(CPD/20) | -0.7563 | 0.3821 | -1.9790 | 0.0478 |  |
| European American*log(CPD/20) | -0.3935 | 0.9747 | -0.4037 | 0.6862 |  |
| ^a^ P values are two-sided from the Wald test. ^b^ Likelihood ratio test p-value comparing to null model with a single term. Abbreviations: BMI - body mass index; CPD - cigarettes per day. | | | | | |

**Supplementary Table 2 Model Statistics for Multiethnic Smoking Associations**

| **Parameter** | **Beta Coefficent** | **Standard Error** | **Test Statistic** | **p-value^a^** |  |
| --- | --- | --- | --- | --- | --- |
| **Log-linear Term 1** |  |  |  |  |  |
| Constant | -8.2590 | 0.1750 | -47.1800 | <0.001 |  |
| Sex |  |  |  |  |  |
| Male | ref |  |  |  |  |
| Female | -0.0997 | 0.0486 | -2.0500 | 0.040 |  |
| Diabetes |  |  |  |  |  |
| No | ref |  |  |  |  |
| Yes | 0.2498 | 0.0704 | 3.5490 | <0.001 |  |
| Race/Ethnicity |  |  |  |  |  |
| African American | ref |  |  |  |  |
| Native Hawaiian | 0.3303 | 0.0972 | 3.3970 | <0.001 |  |
| Latin American | -0.2512 | 0.0786 | -3.1970 | 0.001 |  |
| Japanese American | 0.1176 | 0.0734 | 1.6040 | 0.109 |  |
| European American | -0.2172 | 0.0793 | -2.7390 | 0.006 |  |
| Log(age/70) | 5.2420 | 0.2235 | 23.4600 | <0.001 |  |
| BMI | 0.0202 | 0.0055 | 3.6760 | <0.001 |  |
| **Linear Term 1** |  |  |  |  |  |
| Pack-Years/50 | 0.9071 | 0.1860 | 4.8780 | <0.001 |  |
| **Log-linear Term 2** |  |  |  |  |  |
| Years-Quit | -0.0933 | 0.0353 | -2.6400 | 0.008 |  |
| log(CPD/20) | -0.6186 | 0.2834 | -2.1830 | 0.029 |  |
| ^a^ P values are two-sided from the Wald test. Abbreviations: BMI - body mass index; CPD - cigarettes per day. | | | | | |

**Supplementary Table 3 Sex-Specific Model Results for Smoking Variables**

|  | **Beta (95% CI)** | **Standard Error** | **p** | **HR** |
| --- | --- | --- | --- | --- |
| **Male** |  |  |  |  |
| Pack-years/50 | 0.83 | 0.23 | <0.001 | 1.83 |
| Years-quit | -0.09 | 0.05 | 0.0628 | - |
| Log(CPD/20) | -0.75 | 0.39 | 0.0567 | - |
| **Female** |  |  |  |  |
| Pack-years/50 | 1.04 | 0.31 | <0.001 | 2.04 |
| Years-quit | -0.09 | 0.05 | 0.0647 | - |
| Log(CPD/20) | -0.48 | 0.41 | 0.246 | - |
| Sex-specific models containing all terms from Supplementary Table 2; only smoking variables are shown. P-LRT for sex-pack-years interaction = 0.463. | | | | |


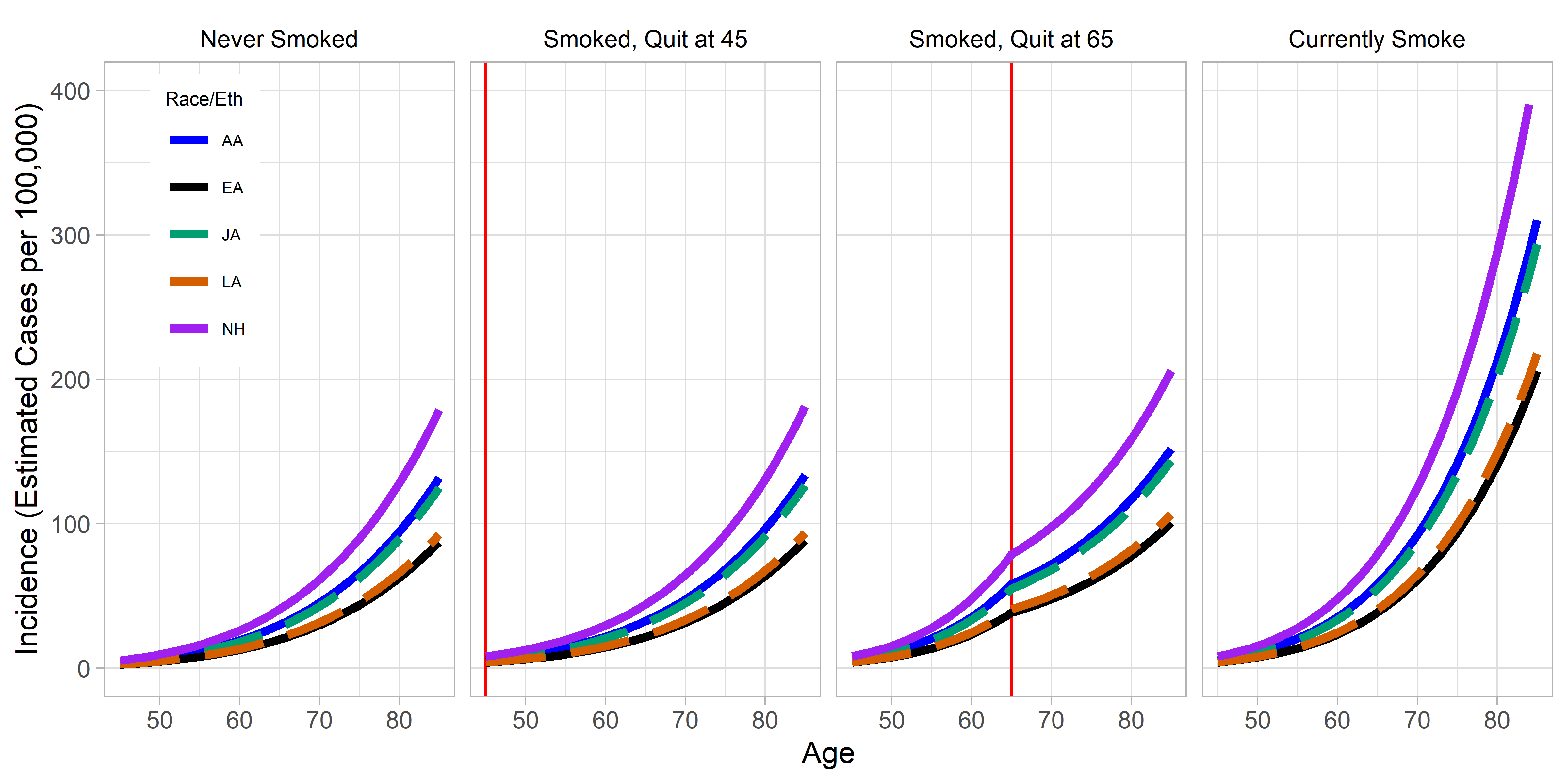


**Supplemental Fig. 1** Predicted risk trajectories among females with differing smoking histories. Risk, in cases per 100,000 (y-axis), is plotted as a function of age (x-axis), pack-years, and years-quit. The vertical red line indicates age at smoking cessation. A simplified model was used to estimate risk in the combined sample (from Supplementary Table 2, fit within sex groups of Supplementary Table 3)


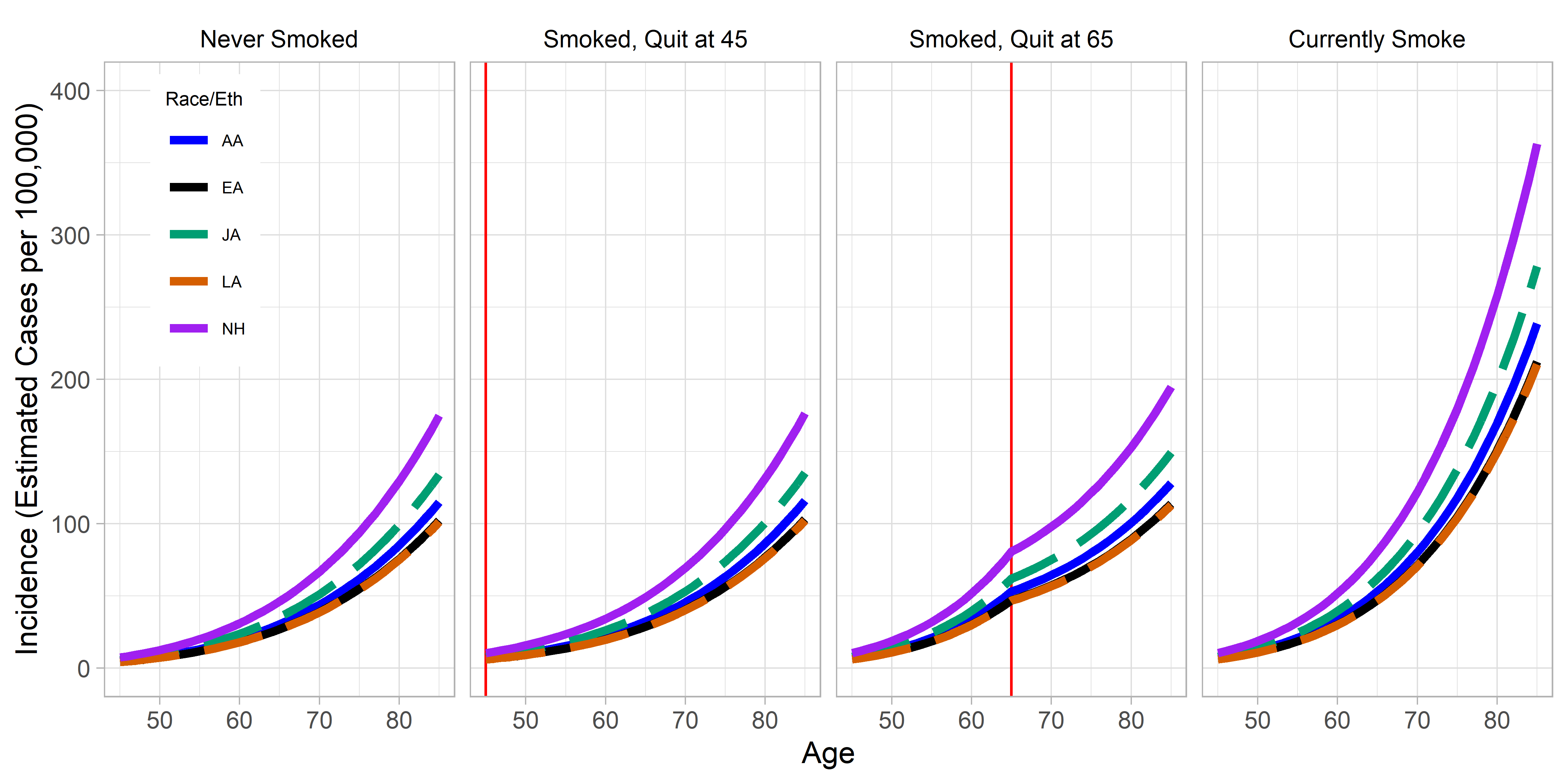


**Supplemental Fig. 2** Predicted risk trajectories among males with differing smoking histories. Risk, in cases per 100,000 (y-axis), is plotted as a function of age (x-axis), pack-years, and years-quit. The vertical red line indicates age at smoking cessation. A simplified model was used to estimate risk in the combined sample (from Supplementary Table 2, fit within sex groups of Supplementary Table 3)
